# Symmetry-Constrained Properties Behave Differently for 2D or 3D Structures under the Same Point Group

**DOI:** 10.1021/acs.jpca.4c02167

**Published:** 2024-05-17

**Authors:** Wagner Eduardo Richter

**Affiliations:** Department of Chemistry, Federal University of Technology − Paraná, Ponta Grossa, Paraná 84.017-220, Brazil

## Abstract

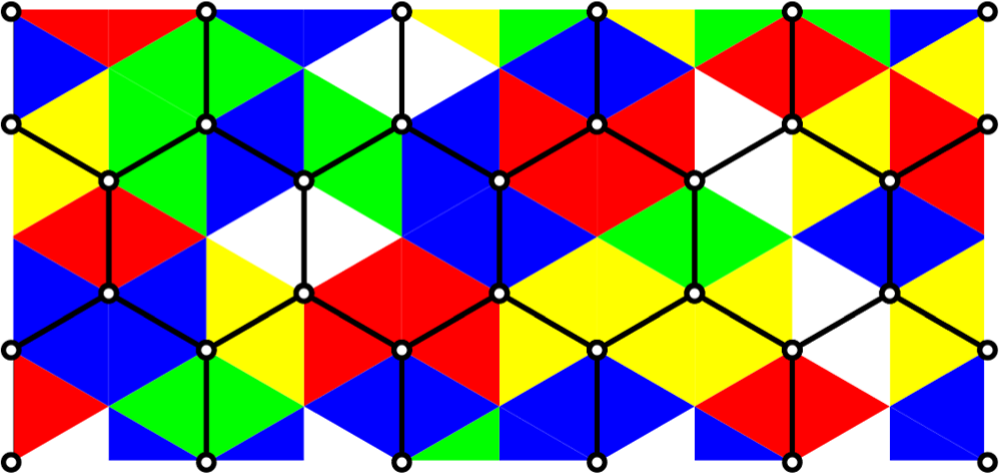

In chemistry and physics, two molecules belonging to the same point group are expected to behave similarly regarding various properties, following their character tables. Here, we show that the derivative of the dipole moment with respect to the normal coordinate of vibration might show different symmetry constraints if the molecule is planar, even if these molecules belong to the same point group. Examples of pairs of molecules featuring these conditions are presented. These findings open a new path toward a much deeper understanding of how 2D materials behave so differently compared to 3D materials featuring the very same atoms and arrangements (graphene and graphite, for example); chemists and physicists dealing with 2D materials could benefit from looking more deeply into pure mathematical relations that might be governing 2D systems in a different way when compared to 3D systems. The aid from mathematicians is welcomed.

## Introduction

From simple isomerism in organic chemistry toward d-orbital splitting in coordination compounds or symmetry-constrained combination of atomic orbitals to build molecular orbitals, symmetry is ubiquitous in nearly all branches of chemistry. Teaching and discussing molecular (or orbital) symmetry usually involves character tables from point groups derived from group theory.^1^ Even though several dozens of these point groups can be derived, just part of them is relevant for chemistry in view of the limited different structures that chemistry allows to be formed.

Not only molecular structures but also molecular properties behave following symmetry-based rules. In this matter, we recall that both infrared (IR) and Raman activities (for molecular vibrations) can be anticipated from the character table of the respective molecule’s point group,^2^ as well as the orientation of dipole^3^ and various other multipole moments,^4^ considerations on melting points,^5^ and so on. In the present report, we want to show a novel finding in that matter: two molecules belonging to the same point group (i.e., they have the same relative internal symmetry) might show different symmetry constraints for molecular properties depending on whether these molecules are two-dimensional (2D, i.e., planar) or three-dimensional (3D) structures. The demonstration will use the *D*_3*h*_ point group as a first example, and once it is ended, other examples can be readily assembled.

## Calculations

All electronic structure calculations were carried out using Gaussian 16^6^ (at the m06-2X/aug-cc-pVTZ level of theory). The structures were optimized and had their standard vibrational analyses at the same level of theory to ensure that they are all stationary points (minimum) in the potential energy surface, with no imaginary frequencies. These structures were then used to produce the CCTDP results, using the program Placzek,^7^ following the very same protocol described in detail elsewhere.^8,9^ Hirshfeld^10,11^ atomic charges and atomic dipoles were used here because of the computational speed (Gaussian delivers these values with little extra computational effort), but other partition schemes (QTAIM,^12,13^ DDEC6^14^ as well as any other featuring atomic dipoles and also satisfying the molecular dipole moment from the wave function^15−17^) would yield different numerical results but absolutely equivalent conclusions.

## Theoretical Background

The property under investigation here is the dipole moment derivative or, more precisely, the derivative of the molecular dipole moment with respect to the normal coordinate of vibration; if the molecular dipole moment is written  and the *k*th normal coordinate (concerning the *k*th molecular vibration) is written *Q*_*k*_, this derivative will then be .^8,18^ When squared and multiplied by the appropriate constants, this derivative yields the IR intensity (*A*_*k*_) of that vibration,^19^ which is also a real, quantitative, and measurable property, and is also easily calculated by most of the standard quantum chemical packages.

The molecular dipole moment is a molecular property, but chemists pursue ways to express molecular properties as sums of atomic properties (just like molar mass is the sum of the atomic masses). Unfortunately, while atomic masses are easily assigned to atoms because they are almost completely concentrated on their respective nuclei (the total mass belonging solely to the electron cloud is negligible), expressing molecular dipole moments in terms of atomic charges is not straightforward^20^ because now the charges from the electrons contribute as much as the charges from the protons at the nuclei. In other words, while a discrete distribution can approximate the set of atomic masses because the mass of the continuous electron cloud might be ignored, the contribution of this continuous electron density to the molecular dipole moment cannot be neglected, so the distribution of charges needs to be taken as a continuous distribution instead.^21^ This has not prevented chemists from using models based solely on simple electric charges positioned at the nuclei, just as eq 1; another approach takes into consideration the inner inhomogeneities of the electron cloud, thus assigning to each atom an intrinsic polarization called atomic dipole, which must be included in the expression for the overall dipole, just like in eq 2(15,16)
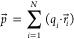
1
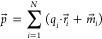
2

When substituting these two into the full derivative given previously, we have, respectively^15,16^
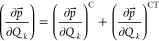
3
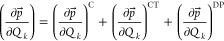
4

The labels on the right-hand side (RHS) of the equations are C (for charge), CT (for charge transfer), and DP (for dipolar polarization).^22^ These terms are interpreted straightforwardly: the charge term regards the effect of the static charges (from the equilibrium geometry) vibrating along the normal coordinate, whereas the charge transfer and dipolar polarization terms concern the changes in the charges and the changes in the atomic dipoles that necessarily occur when the molecular structure departs from the equilibrium arrangement. A comprehensive, detailed, and commented derivation from eqs 1 and 2 toward eqs 3 and 4, respectively, can be found elsewhere.^8,18^

In 1989, Dinur and Hagler explored both these situations^23^ and first noticed that if we are dealing with the out-of-plane bending vibration of a planar molecule (i.e., displacements along the normal coordinate that are perpendicular to the molecular plane), the symmetry of the normal coordinate requires the second derivative in the RHS (the CT one) to vanish, regardless of the charge model being used. This result was used by some authors in the 1990s to pursue the so-called “IR charges”, supposedly derived directly from experiment.^24,25^

In recent years, our group has been exploring this CT = 0 symmetry constraint more detailedly^15−17,26,27^ and discovered that it requires the molecular dipole moment to be expressed as a sum of both atomic charges and atomic dipoles; otherwise the agreement between theoretical and calculated IR intensities (which are real, unambiguous, measurable properties) could not be achieved, regardless of the charge model used. A side result was that the aforementioned IR charges are actually not pure charges but charges contaminated by fluctuations in the atomic dipoles.^15^ In other words, pure atomic charges cannot be obtained directly from experiment, at least not from IR intensity measurements.^18,28^ Dinur and Hagler^23^ had suggested this in their original paper, and we confirmed their suggestions.

Putting it differently, even though atomic charges are not true quantum mechanical observables, these last results from our group demonstrated that atomic charges from models based on a point charge approximation (i.e., the idea that atomic charges only are sufficient for describing the molecular dipole moment) are necessarily incomplete because they cannot capture the overall dynamics of the electron density, while this can be achieved if—and only if—the charge model features atomic dipoles as well.

## Results

Let us take simple planar molecules as examples: formaldehyde (H_2_CO, *C*_2*v*_), boron trifluoride (BF_3_, *D*_3*h*_), ethene (C_2_H_4_, *D*_2*h*_), and benzene (C_6_H_6_, *D*_6*h*_). They are all planar, so all of their out-of-plane vibrations (oopv) will be subjected to the CT = 0 constraint (but not the in-plane vibrations). Given these molecules, we can easily think of a number of examples of nonplanar molecules belonging to the same point groups: maybe the more obvious example would be phosphorus pentafluoride (PF_5_), which is *D*_3*h*_ just as BF_3_; however, since it is not planar, the CT = 0 condition will not be imposed. If both have the same point group, how can they behave differently? Can we explain that at the atomic level?

**Figure 1:**
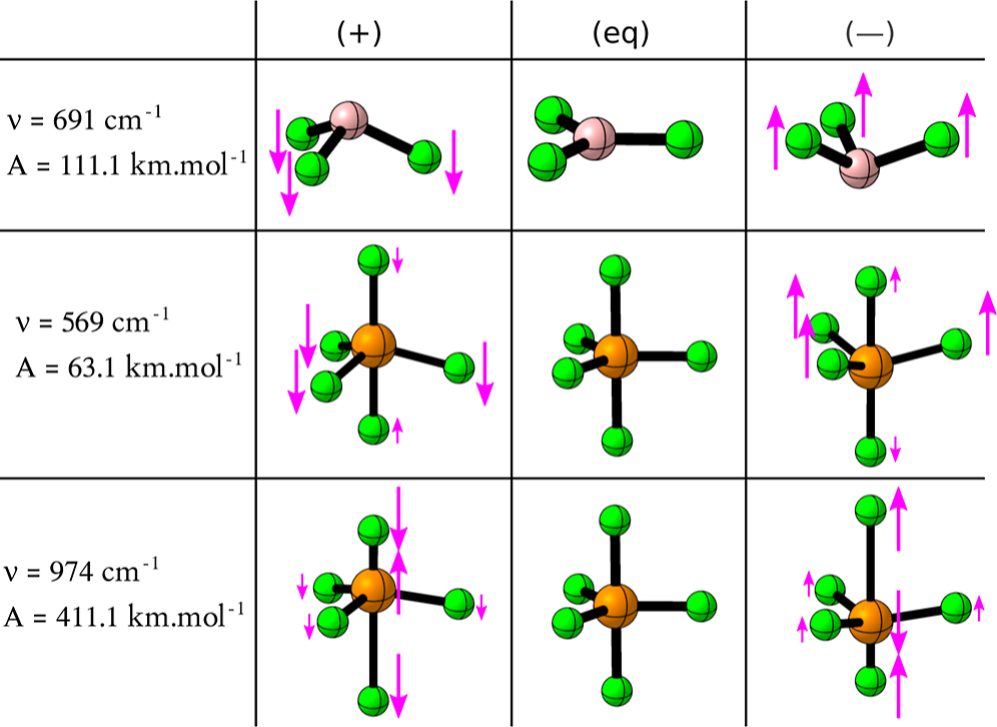
Vibrations in BF_3_ (top) and PF_5_ (middle, bottom), whose atomic displacements are perpendicular to the inner molecular trigonal plane. Frequencies (ν) and IR intensities (*A*) are shown in the first column, calculated using Gaussian 16.^6^ Pink arrows represent the directions of these displacements (approximately to scale). (+) and (−) stand for the positive and negative displacements along the normal coordinate, respectively, whereas (eq) stands for the equilibrium position. Structures were drawn using CYLview^29^ and assembled using Inkscape.

Yes, we can. Let us tackle BF_3_ first, placed along the *xy* plane. The oopv for it will describe displacements of the atoms along the *z* axis, perpendicularly to the molecular plane (Figure 1, top). Regardless of how much charge the boron atom gains (or loses) when moving upward, it necessarily must gain (or lose) the exact same amount when moving downward because the normal coordinate is completely symmetric.^16^ The same holds true for the fluorine atoms. Therefore, the charges might change, because the distorted geometry is different, but the charge derivative will be null because the equilibrium position (the point where the vibration starts) is either a maximum or minimum of the charges with respect to the displacements (see Figures 1 and 2 in ref (16)). The derivative at any point that is either a local maximum or minimum must be zero, so CT = 0, regardless of how these charges were actually computed.

Now, let us take phosphorus pentafluoride, PF_5_, positioned in a way that the equatorial atoms (phosphorus itself plus three of the five fluorine atoms) are positioned in the *xy* plane, whereas the two axial fluorine atoms are placed in positive and negative *z* positions. There are now two different vibrations which are IR-active that only involve atomic displacements along the *z* axis: the angular bending of the equatorial atoms along the *z* direction (Figure 1, middle) and the asymmetric stretch of the axial fluorine atoms (Figure 1, bottom). When both of these vibrations occur, if we look only at the equatorial atoms, it seems that they are experiencing the very same behavior as the atoms in BF_3_, whereas the axial atoms are not. For instance, while one of them is getting closer to the central phosphorus atom, the other is getting farther from it.

These differences between axial and equatorial atoms will reveal themselves when computing the aforementioned IR intensities, following the atomic partition of intensities presented by our group a few years ago.^8^ This protocol aims to divide the total IR intensity (which concerns the entire molecular vibration) into smaller intensity units, with each of them belonging to individual atoms or groups. These individual terms are also subjected to the CCT or CCTDP partitions given in eqs 3 and 4. Here we performed the atomic partition using Hirshfeld charges and dipoles^10,11^ for BF_3_ and PF_5_; the results are presented in Table 1.

**Table 1:** Atomic Partition of IR Intensities of BF_3_ and PF_5_, Computed at the M06-2X/aug-cc-pVTZ Level, Showing That the CT = 0 Constraint Only Applied to the Equatorial Atoms but Not to the Axial Ones^a^

BF_3_^b^	C	CT	DP	total
B	39.50	0.00	75.77	115.27
F	2.54	0.00	–3.91	–1.37
F	2.54	0.00	–3.91	–1.36
F	2.54	0.00	–3.91	–1.36
molecular	47.12	0.00	64.04	111.18

PF_5_^c^	C	CT	DP	total
P	57.06	0.00	39.83	96.89
F_ax_	11.84	120.63	25.32	157.79
F_eq_	0.05	0.00	–0.60	–0.56
F_eq_	0.05	0.00	–0.60	–0.56
F_ax_	11.84	120.63	25.32	157.79
F_eq_	0.05	0.00	–0.65	–0.60
molecular	80.89	241.26	88.62	410.75

PF_5_^d^	C	CT	DP	total
P	13.39	0.00	16.96	30.35
F_ax_	–3.51	17.05	2.04	15.58
F_eq_	2.66	0.00	–2.11	0.54
F_eq_	2.66	0.00	–2.11	0.54
F_ax_	–3.51	17.05	2.04	15.58
F_eq_	2.66	0.00	–2.10	0.55
molecular	14.35	34.10	14.72	63.14

a All values are in km mol^–1^.

b Out-of-plane bending, 691.86 cm^–1^.

c Asymmetric axial stretch, 973.92 cm^–1^.

d Equatorial out-of-plane bending, 569.65 cm^–1^.

From Table 1, one can verify multiple pieces of evidence regarding the respective molecular structures. For instance, the atomic intensities for all fluorine atoms in BF_3_ are equivalent, in agreement with their equivalence in the structure as well as their equivalent displacements along the normal coordinate. For PF_5_, one can see that there are three fluorine atoms that belong to a group that is different from the other two fluorine atoms; the first three are equatorial atoms, thus indeed the atoms within the inner molecular plane, whereas the other two are the axial atoms lying at the perpendicular *z* direction.

Moreover, the CT for the vibration in BF_3_ vanishes not only for the overall (molecular) intensity but also for all the atoms in the molecule because all of them are subjected to the very same constraint. The molecular CT for both vibrations in PF_5_, on the other hand, does not show CT = 0 because the molecule is not planar (2D). The inspection of the atomic intensities will reveal that three of the five fluorine atoms do show CT = 0, while the other two do not. As expected, these three showing CT = 0 are the equatorial atoms, while the axial atoms show CT ≠ 0. We also notice that phosphorus, although approaching one of the axial fluorines, whereas moving away from the other, still shows CT = 0 because its initial position was within the initial inner plane, thus making its charge either a maximum or minimum with respect to the displacement.

**Figure 2:**
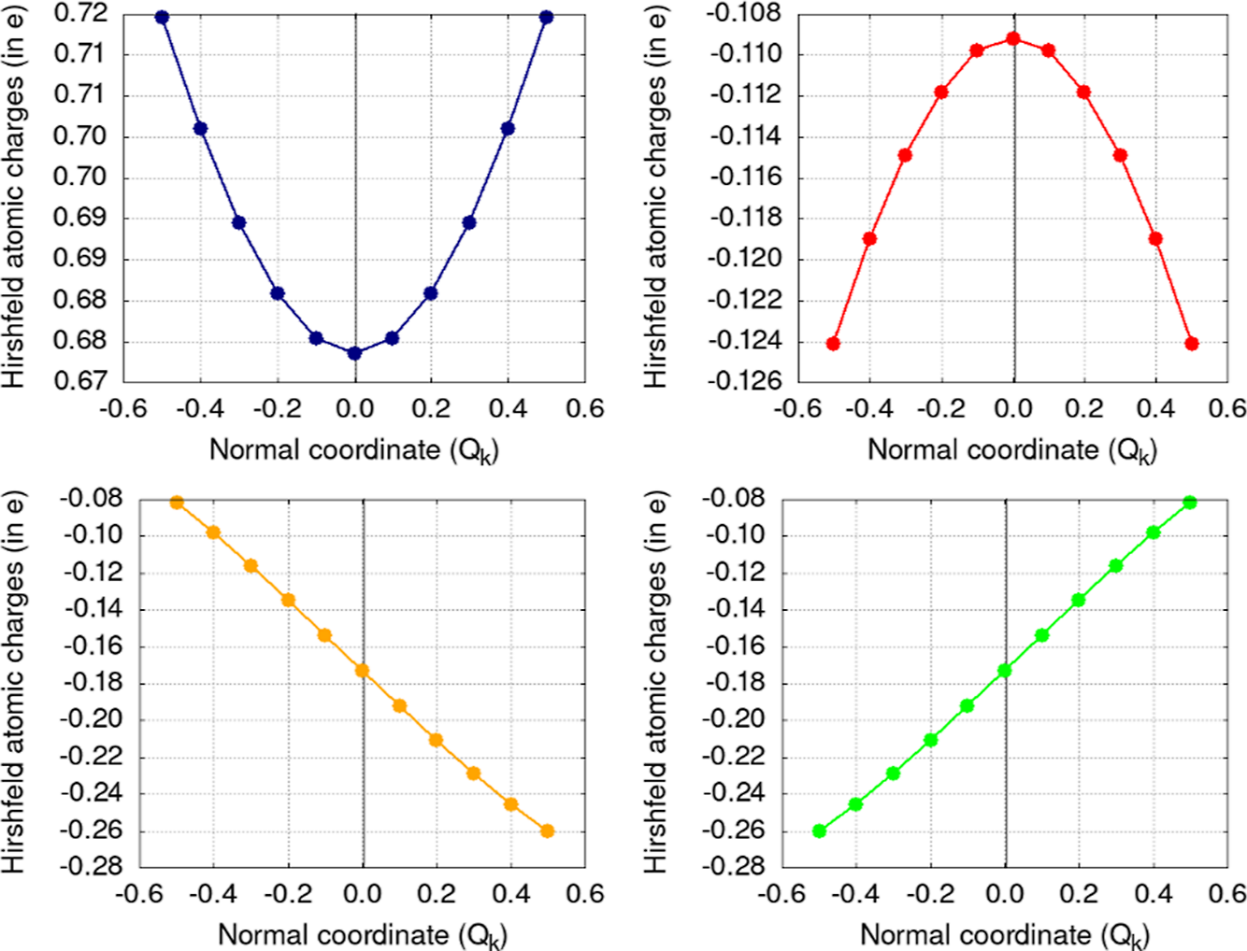
Atomic charges (Hirshfeld partition scheme) for the atoms in PF_5_ for frozen structures obtained from the normal coordinate of vibration: phosphorus (top-left) and the two axial fluorine atoms (bottom-left and bottom-right), plus one of the equatorial fluorine atoms (top-right); the three equatorial atoms show the same behavior.

This can also be demonstrated by evaluating how the atomic charges themselves vary when the molecule follows a normal coordinate. Figure 2 shows the atomic charges of the atoms in PF_5_ along the normal coordinate of the equatorial, out-of-plane bending (Figure 1, top), with the equilibrium position being marked 0.0, at the center, using a black vertical line. Phosphorus (navy line), when moving away from the equilibrium position, will experience an increase in its atomic charge, meaning it is losing electrons to its neighbors. The equatorial fluorine atoms (red line), on the other hand, gain electrons when moving away from the equilibrium position because they become increasingly negatively charged. In both cases, one can see a clear maximum (or minimum) for the function at the equilibrium position, in agreement with CT = 0 (because this CT is indeed a derivative of the charge with respect to the normal coordinate). The axial atoms, on the other hand, experience opposite behaviors: as one of them is getting closer to (farther from) the phosphorus atom, its atomic charge increases (decreases), and the opposite is found for the other atom. As expected, the behaviors for the two axial fluorine atoms are mirror images of one another, and since they show no minimum or maximum at the equilibrium position, their derivatives will not vanish and will account for the CT different from zero in Table 1.

Making a long story short, the CT = 0 symmetry constraint applies only to planar (2D) molecules because this is the only situation in which the atoms are subjected, all together, to the same symmetry constraint. When we have 3D molecules, even though some atoms are still constrained, the atoms that lie outside the plane will behave differently, thus giving to the entire molecule an overall different behavior. This happens because for the planar molecules, the atoms that are equivalent in the equilibrium structure remain equivalent for every distorted geometry that results from the vibration along the normal coordinate. For the 3D molecules, however, atoms that are equivalent at the equilibrium position (the axial atoms, in the above examples) are no longer equivalent in the distorted geometries because one of them is getting closer to the central atom, whereas the other is getting away from it. The initial equivalence is broken. Importantly, we must remember that no molecule remains permanently in the equilibrium position because even at the lowest energy levels available, zero-point vibration will always be present. Therefore, all systems are permanently vibrating and thus subjected to the discussion given here.

## Discussion and Further Insights

Why is that important? Well, previous works from our group used the CCTDP model to evaluate dipole moment derivatives and IR intensities in transition-state structures (TSSs), first using a test set composed of bimolecular nucleophilic substitutions (S_N_2)^30^ and later focusing on the pyramidal inversion of NX_3_ (X = H, F, Cl, and Br) molecules.^31^ If we consider an S_N_2 reaction in which the nucleophile (Nu^–^) and the leaving group (LG^–^) are the same (i.e., H^–^ + H_3_C – H → H – CH_3_ + H^–^), the potential energy surface will be necessarily symmetric with respect to the top of it, which is precisely the position of the TSS.^30^ The same occurs for the pyramidal inversion because the two pyramids (*C*_3*v*_) are equivalent to one another, but the TSS, being planar, is different, belonging to the *D*_3*h*_ point group as well.^31^ Now notice that the TSS of the aforementioned S_N_2 reactions shows the exact same point group (*D*_3*h*_) as the planar TSSs of the pyramidal inversion, but the TSS for the pyramidal inversion is planar, whereas the TSS for the S_N_2 is not. Therefore, the CT term vanishes for the TSS of the pyramidal inversion but does not vanish for the TSS of the S_N_2 reactions.

Indeed, the results for the S_N_2 reactions show that the CT term was, by a large extent, mainly responsible for the overall values of the dipole moment derivatives and their respective IR intensities.^30^ We were expecting that because the mechanism of S_N_2 reactions is indeed described by a net flux of electron density from one reactant (the nucleophile, Nu^–^) to the substrate. However, the TSS of the NX_3_ molecules, because planar, was simply prevented of showing this behavior because being planar automatically implies CT = 0. In other words, we have very similar systems (reactions whose TSSs have the same relative symmetry, *D*_3*h*_), but their electronic behavior is very different because one test set is composed of planar TSSs (subjected to CT = 0) and the other is not. One can think about several other reactions that might be affected by such constraints at their TSSs.

It is worth mentioning that the CT = 0 is a requirement imposed by the equations within the context of planar molecules, and these requirements do not depend on the molecule being treated, as long as they are truly planar.^16,17^ By having this example debunked, various other examples readily came to mind. For example, comparing the out-of-plane bending of a true planar *D*_4*h*_ molecule (like XeF_4_) with the asymmetric stretch in SF_6_, we will see very similar results; since the main axis is defined, the atoms labeled as “equatorial” will show CT = 0, whereas the axial atoms will not. It is true that SF_6_ is not *D*_4*h*_, being *O*_*h*_ instead, but we recall that *D*_4*h*_ is a subgroup inside *O*_*h*_; actually, there are six-coordinate systems which are not completely symmetric (they are *D*_4*h*_ instead of *O*_*h*_) because of Jahn–Teller distortions, and these will also show such patterns. Results for XeF_4_ and SF_6_ are shown in Tables S1 and S2, Supporting Information, while results for *trans-*SCl_2_F_4_ (example of such a distorted octahedron) are shown in Table S3. SF_6_ has another interesting feature: both of the active vibrations are all triply degenerated, and for each of them, one can assign a particular main axis on which two atoms rely on and the other does not. Therefore, for each vibration, we have two atoms belonging to the main axis of the stretch of that vibration, while the other relies on the plane perpendicular to it. As there are three vibrations, the atoms showing CT = 0 or not will be different for each of them, depending on whether they are in the plane or in the main axis. The same occurs for SCl_2_F_4_, but now the vibrations are doubly degenerated (when the four fluorine atoms describe the same bending), whereas the out-of-plane bending of the atoms in the Cl_2_F_2_ plane has no degenerescence.

The aforementioned results allow us to conclude that any molecule featuring such a structure (a few atoms within a plane and other atoms in the axis perpendicular to this plane) will behave in this way, grounding our arguments in nothing more than its equilibrium structure. An insightful example would be IF_7_, which exists as a *D*_5*h*_ structure^32^ having two axial fluorine atoms and five equatorial atoms lying on the same pentagonal plane. Results from PF_5_ and SF_6_ suggest that the five equatorial atoms will show CT = 0, but the axial atoms will not. This is indeed the case, as presented in Table S4, Supporting Information.

Mathematical definitions allow us to go even further than that. We recall that points might be collinear and lines can be coplanar, so if we have two distinct and parallel planes, it is possible to figure out another plane that lies in between those two at equal distances from both. This is precisely the situation for ferrocene, Fe(C_5_H_5_)_2_ [also written Fe(Cp)_2_], a *D*_5*h*_ (if eclipsed) structure just as IF_7_ (but the conclusions also hold for the staggered *D*_5*d*_ structure). If we place each cyclopentadienyl ligand as planes parallel to the *xy* plane (the plane containing the iron atom), then there will be various vibrations featuring just displacements of atoms along the *z* axis. These will not show CT = 0 for the atoms in the ligands because these ligands have no symmetry regarding their own planes. On the other hand, iron will show CT = 0 because its position is one of the infinite points contained in the plane that is equidistant and parallel to both the planes of the ligands. If one wants to have the atoms in the ligand to display CT = 0, we need the ligand as the central plane, which is the case for the triple-decker sandwich complex . The CT = 0 constrains only the ligand at the middle of the structure but not the other ones. Other deck complexes will behave in the same way if one of its parts remains precisely at the plane that divides the entire structure in two mirror images.

The mirror symmetry described in the last paragraph is also important when discussing topological materials,^33^ including topological insulators, semimetals, and others. These have particular topological arrangements in their electronic band structures, causing unconventional electromagnetic behavior.^34^ Although symmetry seems to play an essential role behind these unconventional behaviors, the rational design of topological materials seems to be still unworkable; it was already stated that “*how many topological materials exist, their identity and their abundance is unclear*”^34^ but also that “*the problem underlying the efficient discovery of (topological) materials has been a missing link between the chemistry of different compounds and their topological properties*”.^34^ Such a link is precisely what the present paper aims at. What if, instead of checking whether all the already known materials behave as topological materials or not, we look at symmetry and topological rules that a topological material *must* obey, and then design a material which will *definitely* follow that rule?

One last set of examples comes from surface tiling. It is well-known that pentagons cannot be used to cover a planar surface (periodically), while triangles, squares, and hexagons can. Therefore, molecules such as corannulenes, featuring aromatic rings around a pentagon center, will not be planar on their own (adopting a bowl shape instead), but the TSS that connects the bowl-to-bowl inversion is. This inversion barrier, passing through the planar structure, is significantly lowered when the corannulene is constrained within a molecular cage.^35−37^ The planar corannulene structure, within or without that cage, if vibrating along an out-of-plane normal coordinate, will show CT = 0 because it is symmetric with respect to its own plane, even though the bowl shape will not because it is not planar at all. It is worth remembering that surface tiling also represents a scientific field in which mathematics and chemistry are deeply connected. We recall, for example, the 2011 Nobel Prize in Chemistry for the discovery of quasicrystals that are ordered but not periodic. This quasiperiodic tiling was initially developed within a pure math framework (Penrose tiling^38^ and Wang dominoes^39^) before having its deep impact in applied chemistry,^40^ just as (we hope) the charge-transfer restrictions presented in detail in this manuscript.

## Conclusions

The origin of the astonishing differences between 2D and 3D structures explored in this work seems to be pure mathematical restrictions that are imposed on 2D systems but not on 3D ones, as they do not depend on the nature of the molecule or the atoms involved. It is like a theorem that will hold for any 2D system in nature. For that matter, materials chemists know, for a long time now, that 2D materials behave very differently compared to 3D ones. They show unique features that will not be encountered in any kind of 3D material regardless of its shape or structure, justifying the appearance of scientific journals totally dedicated to 2D systems.

We have demonstrated here that dipole moment derivatives behave differently in 2D and 3D systems solely because mathematical restrictions are imposed by symmetry, but it is certainly possible to speculate now that various other properties might be constrained by symmetry in a similar fashion. With all that in mind, we can build a most valuable insight into the present (and maybe to the future): how much of the various different behaviors observed in materials chemistry rely on pure mathematical constraints similar to the one explored here? Maybe we should increase the efforts in that direction, to comprehensively understand pure “boundary conditions”, based on mathematical restrictions that are imposed on 2D materials but not on 3D ones (and vice versa). Finding other properties subjected to similar constraints would be a major breakthrough for materials chemistry, allowing the ignition of a brand new scientific paradigm for the search of special 2D systems like graphene and other graphene-like nanosheets.^17^ Indeed, we discussed corannulene in the previous paragraphs, and a corannulene-based wavy graphene was reported recently,^41^ while special properties from the lumps in graphene have been reported already 20 years ago.^42^ These properties are directly related to the *planarity: to be or not to be* dilemma.

Measuring properties is still important, but what about gaining much deeper insights into 2D materials by figuring out pure mathematical relationships governing them? This might clear the path toward a more concise and detailed understanding of the unique features of 2D materials, as well as allowing the prediction of properties that would hardly be measured by accident.

We hope that, in future years, various other properties of 2D monolayers and nanosheets can be demonstrated to be rooted in pure mathematical theorems and constraints, and the precise anticipation, identification, and evaluation of these constraints might drive the rational design of new materials with a technological appeal.

## Data Availability

The raw data is made available from the author upon reasonable request. The author also remains available to help anyone interested in reproducing these calculations using the Placzek software.

## Supporting Information Available

Atomic contributions for IR intensities for selected vibrations from XeF_4_, SF_6_, SCl_2_F_4_, and IF_7_, presented in the same format of Table 1 (PDF)
