# Supplementary material for: Symmetry-Constrained Properties Behave Differently for 2D or 3D Structures under the Same Point Group
Source: J Phys Chem A. 2024 May 17;128(21):4308–14. doi: 10.1021/acs.jpca.4c02167 (PMC11145645; doi:10.1021/acs.jpca.4c02167)
Supplement: Supplementary file 1 — jp4c02167_si_001.pdf [file jp4c02167_si_001.pdf]

Supporting Information

"Symmetry Constrained Properties Behave Differently For 2D Or 3D Structures Under The Same Point Group"

Wagner Eduardo Richter*

Department of Chemistry, Federal University of Technology – Paraná.

Ponta Grossa, PR, Brazil. Postal Code 84.017-220.

E-mail: richter@utfpr.edu.br

Table S1: Atomic partition of infrared intensities of XeF_4, computed at the M06–2X/def2TZVP level, showing that the $CT = 0$ constraint only applied to the equatorial atoms, but not for the axial ones. All values in $km\ mol^{-1}$.

XeF_4, 316.09[a]	C	CT	DP	Total
Xe	10.84	0.00	16.83	27.67
F	4.70	0.00	-0.36	4.34
F	4.70	0.00	-0.36	4.34
F	4.70	0.00	-0.36	4.34
F	4.70	0.00	-0.36	4.34
Molecular	29.64	0.00	15.39	45.03

[a] out–of–plane bending, 316.09 cm^{-1}, $A = 44.03\ km\ mol^{-1}$.

Table S2: Atomic partition of infrared intensities of SF_6, computed at the M06–2X/def2TZVP level, showing that the $CT = 0$ constraint only applied to the equatorial atoms, but not for the axial ones. All values in $km\ mol^{-1}$.

SF_6, 992.53[a]	C	CT	DP	Total
S	61.37	0.00	35.37	96.74
F	0.69	0.00	-0.74	-0.05
F	0.69	0.00	-0.74	-0.05
F	7.23	153.63	18.92	179.77
F	0.69	0.00	-0.74	-0.05
F	0.69	0.00	-0.74	-0.05
F	7.23	153.63	18.92	179.77
Molecular	78.59	307.26	70.25	456.08
SF_6, 621.26[b]	C	CT	DP	Total
S	8.55	0.00	10.07	18.62
F	1.60	0.00	-1.43	0.17
F	1.60	0.00	-1.43	0.17
F	-2.00	8.81	1.03	7.84
F	1.60	0.00	-1.43	0.17
F	1.60	0.00	-1.43	0.17
F	-2.00	8.81	1.03	7.84
Molecular	10.95	17.62	6.41	34.98

[a] asymmetric axial stretch, 992.53 cm^{-1}, $A = 456.07\ km\ mol^{-1}$.
[b] out–of–plane bending, 365.50 cm^{-1}, $A = 35.00\ km\ mol^{-1}$.

Table S3: Atomic partition of infrared intensities of SCl_2F_4, computed at the M06–2X/def2TZVP level, showing that the $CT = 0$ constraint only applied to the equatorial atoms, but not for the axial ones. All values in $km\ mol^{-1}$.

924.42[a]	C	CT	DP	Total
S	42.39	0.00	23.75	66.15
F	0.58	0.00	-0.45	0.13
F	6.59	121.09	14.46	142.14
F	0.58	0.00	-0.45	0.13
F	6.59	121.09	14.46	142.14
Cl	0.02	0.00	-15.84	-15.82
Cl	0.02	0.00	-15.84	-15.82
Molecular	56.77	242.18	20.09	319.05
687.95[b]	C	CT	DP	Total
S	73.84	0.00	26.93	100.77
F	4.60	0.00	-4.14	0.45
F	4.60	0.00	-4.14	0.45
F	4.60	0.00	-4.14	0.45
F	4.60	0.00	-4.14	0.45
Cl	0.86	267.43	37.62	305.92
Cl	0.86	267.43	37.62	305.92
Molecular	93.96	534.86	85.61	714.41
498.35[c]	C	CT	DP	Total
S	4.01	0.00	3.50	7.51
F	0.15	1.45	-0.14	1.47
F	0.07	1.57	-0.10	1.54
F	0.15	1.45	-0.14	1.47
F	0.07	1.57	-0.10	1.54
Cl	0.12	0.00	-2.08	-1.97
Cl	0.12	0.00	-2.08	-1.97
Molecular	4.69	6.04	-1.14	9.59
461.49[d]	C	CT	DP	Total
S	0.54	0.00	-0.79	-0.25
F	-0.28	0.00	0.17	-0.11
F	-0.28	0.00	0.17	-0.11
F	-0.28	0.00	0.17	-0.11
F	-0.28	0.00	0.17	-0.11
Cl	0.17	0.94	-0.15	0.96
Cl	0.17	0.94	-0.15	0.96
Molecular	-0.24	1.88	-0.41	1.23

[a] asymmetric F–S–F axial stretch, 924.42 cm^{-1}, $A = 318.22\ km\ mol^{-1}$, doubly degenerated.
[b] asymmetric Cl–S–Cl axial stretch, 687.95 cm^{-1}, $A = 715.56\ km\ mol^{-1}$.
[c] out–of–plane [Cl_2F_2 plane] bending, 498.35 cm^{-1}, $A = 9.52\ km\ mol^{-1}$, doubly degenerated.
[d] out–of–plane [F_4 plane] bending, 461.49 cm^{-1}, $A = 1.16\ km\ mol^{-1}$.

Table S4: Atomic partition of infrared intensities of IF_7, computed at the M06–2X/def2TZVP level, showing that the $CT = 0$ constraint only applied to the equatorial atoms, but not for the axial ones. All values in $km\ mol^{-1}$.

IF_7, 806.39[a]	C	CT	DP	Total
I	18.07	0.00	5.48	23.54
F	0.16	0.00	-0.44	-0.28
F	0.16	0.00	-0.44	-0.28
F	5.17	58.38	2.25	65.80
F	0.16	0.00	-0.44	-0.28
F	0.16	0.00	-0.45	-0.29
F	0.16	0.00	-0.45	-0.29
F	5.17	58.38	2.25	65.80
Molecular	29.21	116.76	7.76	153.72
IF_7, 365.50[b]	C	CT	DP	Total
I	11.32	0.00	18.33	29.65
F	3.32	0.00	-1.09	2.23
F	3.32	0.00	-1.09	2.23
F	-1.13	8.11	0.47	7.45
F	3.32	0.00	-1.09	2.23
F	3.32	0.00	-1.09	2.23
F	3.32	0.00	-1.09	2.23
F	-1.13	8.11	0.47	7.45
Molecular	25.66	16.22	13.82	55.70

[a] asymmetric axial stretch, 806.39 cm^{-1}, $A = 153.72\ km\ mol^{-1}$.
[b] out–of–plane bending, 365.50 cm^{-1}, $A = 55.69\ km\ mol^{-1}$.
